# An Infrared Energy Device for the Treatment of Facial Skin Aging

**DOI:** 10.3390/biomedicines13122878

**Published:** 2025-11-25

**Authors:** Paweł Kubik, Wojciech Gruszczyński, Aleksandra Pawłowska, Maciej Malinowski, Brygida Baran, Agnieszka Pawłowska-Kubik, Łukasz Kodłubański, Bartłomiej Łukasik

**Affiliations:** 1 K-LAB Badania i Rozwój, 81-312 Gdynia, Poland; wojciech.gruszczynski@k-lab.com.pl (W.G.); aleksandra.pawlowska@k-lab.com.pl (A.P.); maciejmal@gmail.com (M.M.); agnieszkapawl@wp.pl (A.P.-K.); 2 Medical Department, Matex Lab Switzerland SA, 1228 Geneve, Switzerland; brygida.baran@neauvia.com (B.B.); bartlomiej.lukasik@neauvia.com (B.Ł.); 3 Department of Human Rights and Intellectual Property Law, University of Gdansk, 80-309 Gdansk, Poland; lukasz.kod@gmail.com

**Keywords:** infrared energy device, facial skin aging, facial skin tightening, thermolifting, esthetic treatment, collagen stimulation

## Abstract

**Objectives:** To evaluate the cellular and clinical responses to facial treatment using a wide-spectrum, long-pulse infrared medical device. **Methods:** Thirty patients with facial skin laxity and photodamage underwent a single treatment session with the infrared device. Skin biopsies were collected before treatment and on days 5 and 21 for histological and immunohistochemical assessments of inflammatory and regenerative changes. Long-term outcomes were analyzed by biomechanical measurements (cutometry) and collagen autofluorescence quantification from histological samples. **Results:** Histological analysis demonstrated a 40% increase in fibroblast count, angiogenesis, and mild inflammatory activity, though these changes did not reach statistical significance. Cutometric evaluation revealed a significant improvement in skin elasticity at day 21 (+15.97%) and day 150 (+24.49%) post-treatment, accompanied by a modest increase in hydration. Collagen autofluorescence showed a statistically significant enhancement, consistent with ECM remodeling and fibroblast activation. **Conclusions:** A single session of treatment with a wide-spectrum, long-pulse infrared device produced measurable improvements in dermal elasticity and collagen organization with minimal inflammatory response. These findings support the efficacy and safety of infrared energy-based therapy as a noninvasive modality for facial skin rejuvenation.

## 1. Introduction

Facial skin is continuously exposed to both intrinsic and extrinsic aging processes, resulting in structural and functional changes that contribute to visible signs of aging such as laxity, fine lines, and wrinkles. Intrinsic aging, driven largely by genetic and hormonal factors, leads to progressive dermal thinning and reduced fibroblast activity, whereas extrinsic factors, including chronic ultraviolet (UV) radiation, pollution, and lifestyle influences, exacerbate oxidative stress and accelerate collagen degradation. Collectively, these mechanisms diminish dermal elasticity and hydration, resulting in clinically evident photodamage and loss of skin resilience [1,2]. Beyond esthetic concerns, these changes also have functional implications, including impaired barrier function, reduced biomechanical strength, and altered cutaneous physiology.

A growing body of research has focused on noninvasive energy-based technologies as alternatives to surgical interventions for skin rejuvenation. Among these, infrared (IR) devices have gained particular interest due to their ability to penetrate deeply into the dermis, where they induce controlled thermal effects [3]. This process stimulates neocollagenesis, angiogenesis, and remodeling of extracellular matrix components, ultimately improving tissue elasticity and clinical appearance [3,4,5,6]. Unlike ablative modalities such as CO_2_ or Er:YAG lasers, which involve epidermal disruption and longer recovery times, infrared-based devices can achieve favorable regenerative outcomes with minimal downtime and a favorable safety profile [5,7,8].

In addition to energy-based modalities, numerous topical and minimally invasive approaches are employed to counteract facial aging. Topical agents such as retinoids, antioxidants, peptides, and growth-factor-enriched formulations remain foundational due to their ability to enhance epidermal turnover, reduce oxidative stress, and support dermal repair [2,9,10]. Alongside these methods, a wide range of injectable techniques has been developed to address dermal atrophy, extracellular matrix disorganization, and soft-tissue volume loss. Injectable treatments, including hyaluronic acid derivatives, collagen-stimulating agents, amino acid–enriched formulations, choline-based solutions, and other biologically active compounds, have demonstrated efficacy in improving hydration, elasticity, and overall skin quality [11,12]. Recent studies show that mesotherapy with low–molecular-weight hyaluronic acid fragments or combinations of hyaluronic acid and choline enhances collagen production, angiogenesis, and epidermal thickness, contributing to visible rejuvenation and high patient satisfaction [13,14,15]. Additionally, agents such as botulinum toxin, deoxycholic acid, and synthetic fillers further expand therapeutic possibilities for dynamic wrinkles, localized adiposity, and structural deficiencies [11]. Taken together, these modalities highlight the multimodal nature of contemporary facial rejuvenation, integrating topical treatments, injectables, and energy-based devices tailored to individual clinical and anatomical needs.

The infrared thermolifting device evaluated in the present study represents a class of non-invasive technologies designed to improve skin firmness, elasticity, and overall facial appearance through controlled dermal heating. By delivering long-pulse infrared energy to deeper skin layers, the device induces a progressive increase in dermal temperature that leads to immediate collagen fiber contraction and stimulates fibroblast activity. These thermal effects activate neocollagenesis and promote remodeling of the extracellular matrix, resulting in enhanced tissue tension, improved skin quality, and visible reductions in early signs of facial laxity [16]. Clinical outcomes reported for infrared thermolifting include improvements in cutometric skin elasticity, smoother surface texture, and high patient satisfaction. However, despite these documented functional and esthetic benefits, comprehensive biological evidence explaining these effects remains limited. Existing studies provide only partial or preliminary insight into the histological and immunohistochemical changes that accompany infrared-based dermal remodeling. Because the present work specifically investigates tissue responses induced by this infrared device, a deeper understanding of the underlying cellular mechanisms is essential for optimizing treatment parameters and advancing evidence-based clinical application [4,16].

In this study, we sought to evaluate both the short-term histological and immunohistochemical responses, as well as the long-term clinical outcomes, of treatment with a wide-spectrum, long-pulse infrared medical device in patients with facial skin laxity and photodamage. By combining quantitative tissue analyses with biomechanical and optical assessments, we aimed to provide an integrated view of the regenerative processes initiated by infrared therapy.

## 2. Material and Methods

### 2.1. Device Used in the Study

The Zaffiro system (Zaffiro Z200NG, Berger & Kraft Medical Sp. z o.o., Warsaw, Poland) is a CE-certified medical device designed for non-invasive skin tightening and rejuvenation using a combination of water-peeling technology and long-pulse broad-spectrum infrared (IR) irradiation. The device delivers controlled IR energy through a sapphire-glass applicator, enabling uniform thermal penetration into the dermis. A preparatory water-peeling module provides precise epidermal exfoliation and improves thermal conductivity, promoting consistent heating of the target structures. Its integrated sapphire cooling system allows safe energy delivery while protecting the epidermis, minimizing discomfort and reducing recovery time [16].

### 2.2. Participants and Exclusion Criteria

Thirty patients (25 women and 5 men), aged 25–68 years (mean age 47.1 years) with facial skin laxity and photodamage, and Fitzpatrick skin phototypes I–IV, were enrolled in the study. Patients underwent the treatment protocol described below.

Exclusion criteria included:

- Use of oral and/or topical retinoids within the last 6 months.
- Excessive tanning.
- Active skin or connective tissue diseases associated with photosensitivity (e.g., systemic lupus erythematosus, porphyria cutanea tarda).
- Active herpes simplex infection.
- Use of drugs or photoreactive cosmetics within the last 6 months, including:
  ▪ Tetracycline antibiotics.
  ▪ Immunosuppressive agents (e.g., corticosteroids and derivatives).
  ▪ Anticoagulants (e.g., dipyridamole, coumarin derivatives).
  ▪ Cosmetics containing thyme extract or herbal products such as St. John’s wort.
- Immunodeficiency disorders (including active HIV infection).
- Fitzpatrick skin phototype VI.
- Pregnancy (as a precaution).
- Uncontrolled diabetes mellitus.
- Previous cosmetic or esthetic procedures in the treatment area (eligibility determined by the physician depending on the procedure performed).
- Acquired vitiligo or other disorders of melanin production (e.g., hypermelanosis).
- Tattoos in the areas designated for treatment.
- Use of anti-inflammatory medications.

Each patient signed an informed consent. The leading standard adopted in this study was ISO 14155:2020 (clinical investigation of medical devices for human subjects—good clinical practice). The study was conducted in accordance with the Declaration of Helsinki, and approved by the Ethics Committee of Medical Chamber in Gdańsk, Poland (protocol code: 2/MATEX/2022; 6 December 2022).

### 2.3. Treatment Protocol

Day 0: All patients were treated with a wide-spectrum infrared device applied to the entire face, including the area behind the ears (lower retroauricular regions on both sides). The treatment utilized a wavelength range of 750–1800 nm with a fluence of 35–45 J/cm^2^.

### 2.4. Assessment Protocol

Day 0: Before the treatment, all patients underwent cutometric tests regarding skin elasticity and hydration. The measurements were made on cheeks (in the area of the middle part of the right or left cheek (randomly selected) at intervals of about 1 cm). In addition, a subgroup of 3 patients was randomly selected for histological evaluation. In these patients, skin and subcutaneous tissue samples (taken by surgical excision) were collected by a team of surgical and dermatological specialists experienced in collecting material for histopathological evaluation of treatment safety and efficacy in esthetic procedures using medical devices. The material at day 0 was collected in the area behind the ear in the lower retroauricular region (right side, behind the *Lobulus auriculae.*

Day 5: All patients underwent cutometric tests regarding skin elasticity and hydration. The measurements were made on both cheeks, three measurements for each cheek. In addition, skin and subcutaneous tissue samples were collected from the subgroup of 3 patients who had been selected at Day 0 for histological evaluation, using surgical excision. The material at day 5 was collected in the area behind the ear in the lower retroauricular region (left side, behind the *Lobulus auriculae*). Samples were collected from the contralateral side of the face to avoid interference with tissue healing from the Day 0 biopsy.

Day 21: All patients underwent cutometric tests regarding skin elasticity and hydration. The measurements were made on both cheeks, three measurements for each cheek. In addition, skin and subcutaneous tissue samples were collected from the subgroup of 3 patients selected at Day 0 for histological evaluation, using surgical excision. Biopsies were obtained from the lower retroauricular region (right side, behind the earlobe) at least 2 cm away from the Day 0 sampling site to avoid interference from ongoing tissue repair processes.

Day 150: All patients underwent cutometric tests regarding skin elasticity and hydration. The measurements were made on both cheeks, three measurements for each cheek. In a subgroup of 3 patients selected at Day 0 for histological evaluation, skin samples were collected using shallow surgical excision. Biopsies were obtained from the lower retroauricular region (left side, behind the earlobe) at least 2 cm away from the Day 5 biopsy site to avoid interference from tissue repair processes. Sampling was limited to the skin layer, as subcutaneous tissue is not assessed in collagen autofluorescence analysis.

### 2.5. Assessment Methods

A. Clinical assessment

Cutometric evaluation

All patients underwent cutometric measurements regarding skin elasticity and hydration. The cutometric evaluation was carried out using the Courage + Khazaka Multi Skin Test Center MC1000 device in standardized conditions of temperature (22–23 °C) and air humidity (55–60%). The procedure of patient preparation for the examination was also standardized (make-up was removed at least one hour before measurement without using alcohol-based products). Measurements were performed by properly trained personnel on the skin of the buccal region. Skin elasticity and hydration were assessed on a 1–100 scale, where 1 represents the worst and 100 the best outcome. Three point-by-point measurements were obtained for each patient, and the average values were recorded in the observation chart for subsequent statistical analysis.

Healing time observation

**Figure 1:**
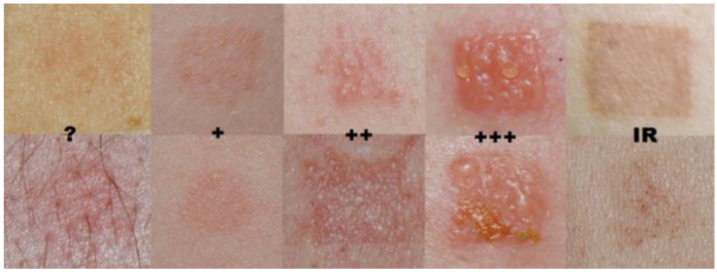
**Visualization of the International Contact Dermatitis Research Group (ICDRG) scale provided to participants.** To enhance scientific clarity, dermatological terminology has been standardized in accordance with ICDRG guidelines. **Negative reaction:** No visible or palpable change. (?) **Doubtful reaction:** Subtle erythema, non-palpable erythematous macule; such a response is generally not considered evidence of sensitization. (+) **Weak reaction:** Palpable erythema suggesting mild edema or infiltration, with or without small papules; no vesicles. (++) **Strong reaction:** Pronounced edema and infiltration with papules and distinct vesicles. (+++) **Extreme reaction:** Coalescing vesicles or bullae, occasionally accompanied by superficial erosions. (IR) **Irritant reaction:** Non-allergic response characterized by shiny or dry skin, erythema, or fine papules.

The assessment of healing time was based on patient self-observation. Two parameters were evaluated: the duration of post-procedural redness and swelling. To minimize subjectivity and standardize reporting, patients were provided with visual reference guidelines adapted from the International Contact Dermatitis Research Group (ICDRG) scale (Figure 1). Although originally developed for evaluating reactions in patch testing for contact dermatitis, the ICDRG scale was selected as a structured, clinical-grade visual tool suitable for guiding consistent self-assessment of transient, mild skin responses. In this study, the scale was used exclusively to support standardized description of short-term erythema rather than for diagnostic purposes. This adaptation strategy allowed participants to classify visible changes more reliably, while acknowledging that the ICDRG scale has not been formally validated for esthetic self-assessment.

Graphical scale of the ICDGR test reading:

Patient’s satisfaction evaluation

The patients’ satisfaction was assessed on the basis of a survey conducted five months after the procedure. Participants were asked to evaluate the results in terms of skin tightening, reduction in fine wrinkles, changes in skin texture and changes in skin color. Each of the above features was assessed by patients on a five-point scale:

(1): No improvement;
(2): Poor improvement;
(3): Moderate improvement;
(4): Good improvement;
(5): Very good improvement.

Expert’s evaluation

The expert assessment was carried out by an independent team of seven researchers, each with at least five years of experience in dermatology and/or esthetic medicine. The experts were shown photographs of patients taken immediately before and five months after the procedure. They were blinded to the type of treatment administered. Assessment was performed via an anonymous survey, in which experts evaluated changes in skin tension, fine wrinkles, skin texture, and skin color. Each parameter was rated on a five-point scale:

(1): No improvement;
(2): Poor improvement;
(3): Moderate improvement;
(4): Good improvement;
(5): Very good improvement.

B. Histological assessment

For histological evaluation, three patients were randomly selected for biopsy. The limited number of biopsies reflected a deliberate methodological choice aimed at minimizing invasiveness and patient burden in this pilot study. Full-thickness skin and subcutaneous tissue samples were collected from the retroauricular region at days 0, 5, and 21 to assess collagen content, fibroblast density, neoangiogenesis, and the intensity of procedure-induced inflammation. At day 150, a skin-only biopsy was obtained to evaluate long-term collagen remodeling. The retroauricular area was chosen to avoid visible scarring and to maintain ethical acceptability of repeated sampling. All procedures were conducted under sterile conditions with local anesthesia (lidocaine). To reduce the potential influence of prior biopsies on subsequent tissue responses, samples were taken from anatomically adjacent but non-overlapping sites within the treated region.

Basic staining

Skin samples were fixed for 24 h in neutral 10% buffered formalin (pH 7.2) and processed using an automated histoprocessor (Excelsior™ AS; Thermo Fisher Scientific, Waltham, MA, USA), following standard pathology protocols. The tissue blocks were sectioned at 5 μm for histological analysis and 3–4 μm for immunohistochemistry. Hematoxylin and eosin (H&E) staining was used to evaluate the cellular content and overall tissue architecture. Masson’s trichrome staining (Special Stain Kit Masson’s Trichrome, DiaPath, Martinengo, Italy) was performed to assess collagen structure.

Immunohistochemistry

The immunohistochemical (IHC) staining was performed in the automated slide-processing system BenchMark^®^ ULTRA (Roche Diagnostics/Ventana Medical Systems, Tucson, AZ, USA) with the ultraView Universal DAB Detection Kit (Roche Diagnostics/Ventana). The following primary antibodies were applied: mouse monoclonal anti-CD68 (KP-1), rabbit monoclonal anti-CD8 (SP57), mouse monoclonal anti-CD20 (L26), mouse monoclonal anti-CD31 (JC70) and mouse monoclonal anti-Vimentin (V9).

Briefly, after deparaffinization and rehydration, heat-induced epitope retrieval was carried out in the Cell Conditioning 1 solution (CC1; Ventana, Camperdown, NSW, Australia) at 72 °C for 68 min. Endogenous peroxidase activity and nonspecific binding sites were blocked by incubating tissue sections with 3% H_2_O_2_ for 10 min at room temperature (RT) and 3% bovine serum albumin (BSA) for 15 min at RT, respectively. Following incubation with primary antibodies and visualization of protein-antibody complexes, tissue sections were counterstained with Mayer’s hematoxylin and bluing reagent, dehydrated, cleared in xylene, and cover-slipped with Dako mounting medium (Agilent Technologies, Santa Clara, CA, USA). Known positive control sections were used for each antibody according to the datasheet, while negative controls were obtained by omitting the primary antibody.

Staining evaluation

Protein expression was evaluated at 10× and 20× magnification, using a Nikon ECLIPSE E400 light microscope (Nikon Instruments Europe, Amsterdam, The Netherlands) by two independent pathologists. Inflammatory infiltration was assessed on H&E stained tissue sections by the percent of area infiltrated by inflammatory cells, including mononuclear cells. A five-point (0–4) grading system was used:

- 0: not detected;
- 1: up to 25% infiltration;
- 2: 26–49% infiltration;
- 3: 50–75% infiltration;
- 4: 76–100% infiltration.

Collagen structure was evaluated on Masson’s trichrome-stained sections (Special Stain Kit Masson’s Trichrome, DiaPath, Martinengo, Italy) using the following scale:

- 0: loose, regular fibers;
- 1: loose, irregular fibers;
- 2: dense fibers;
- 3: compact, coarse fibers.

Expression of CD4 [17], CD8 [18], CD68 [19] proteins was assessed as the percentage of positive inflammatory cells (0–100%).

CD20 [20] expression was scored on a binary scale (0–1). Vascularization was assessed by CD31 [21] staining using the following scale:

- 0: few, thin-walled vessels;
- 1: increased number of vessels with slight wall thickening;
- 2: increased number of vessels with pronounced wall thickening.

The presence of fibroblasts was semi-quantitatively assessed using Vimentin [22] staining on a 0–4 scale.

Autofluorescence analysis of unstained preparations

The analysis of subepidermal collagen autofluorescence was performed on unstained tissue sections using a Nikon C1 confocal microscope with the red channel (543 nm laser excitation, 650 nm long-pass emission filter) and a 20× objective. Image acquisition parameters were kept constant across all samples. Three images were acquired from each section at a resolution of 1024 × 1024 px. Autofluorescence intensity was measured at five points within the ROI, each with dimensions of 80 × 80 px.

Statistical analysis

Statistical analysis of skin elasticity and hydration measurements was performed using IBM SPSS Statistics software (version 28.0.1.0; 2021). Mean values were compared using a paired *t*-test. For hypothesis testing, the following definitions were adopted:

- Null hypothesis: the treatment has no effect on the tested parameters.
- Alternative hypothesis: the treatment significantly affects the tested parameters.

Statistical analysis of histopathological results was performed using GraphPad Prism software (ver. 10.0.2; 2023). Tukey’s multiple comparisons test was used for evaluating differences between groups.

## 3. Results

A. Results of clinical assessment

Cutometric evaluation

1. Skin elasticity measurements

**Figure 2:**
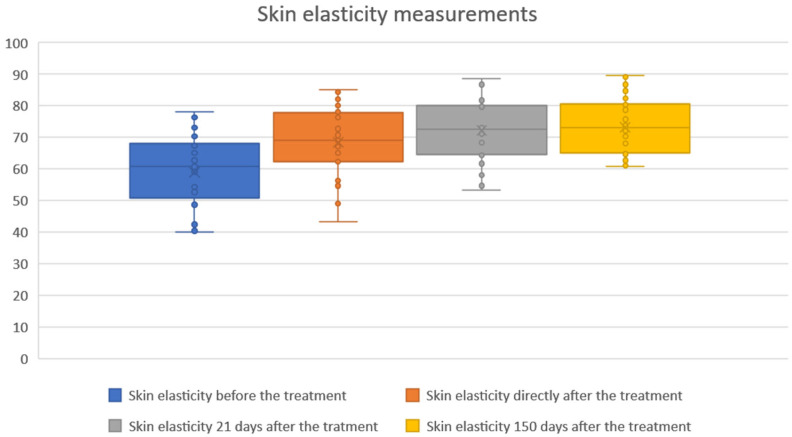
Skin elasticity measurements before and after treatment. Boxplots represent skin elasticity values measured at four time points: baseline (before treatment), immediately after treatment, 21 days after treatment, and 150 days after treatment. An increase in both median and overall distribution of skin elasticity was observed directly after the procedure and maintained throughout the follow-up period. The results indicate a sustained and progressive improvement in skin elasticity over time.

**Figure 3:**
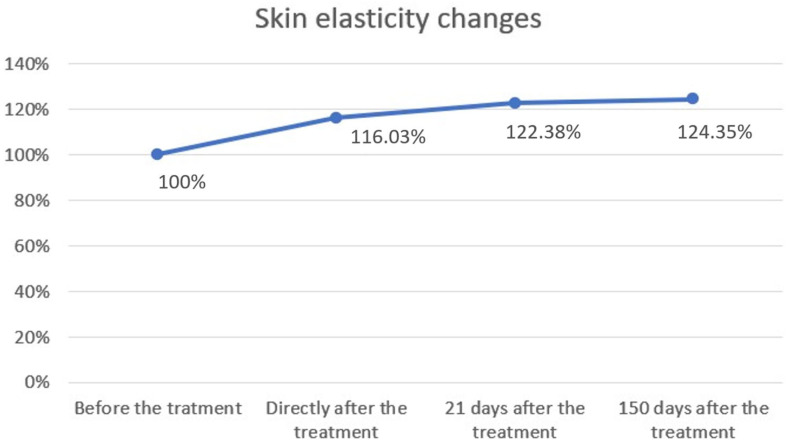
Skin elasticity changes over time. Skin elasticity was measured before treatment, immediately after treatment, and at follow-up visits on days 21 and 150. Baseline values were normalized to 100%. A progressive increase in elasticity was observed, rising to 116.03% immediately after treatment, 122.38% after 21 days, and 124.35% after 150 days. These results demonstrate a sustained improvement in skin elasticity following the procedure.

The study showed statistical significance and a positive correlation of the effect on changes in skin elasticity at 5, 21 and 150 days after the procedure. The effect was observed soon after the procedure and showed gradual improvement over time. The mean elasticity values were as follows: baseline, 58.92; 5 days after the procedure 68.37 (+16.03% increase from baseline), 21 days after the procedure 72.11 (+22.38% increase) and 150 days after the procedure 73.27 (+24.35% increase). The statistical analyses and corresponding graphical results are shown in the tables and figures below (Table 1; Figure 2 and Figure 3).

2. Skin hydration measurements

**Figure 4:**
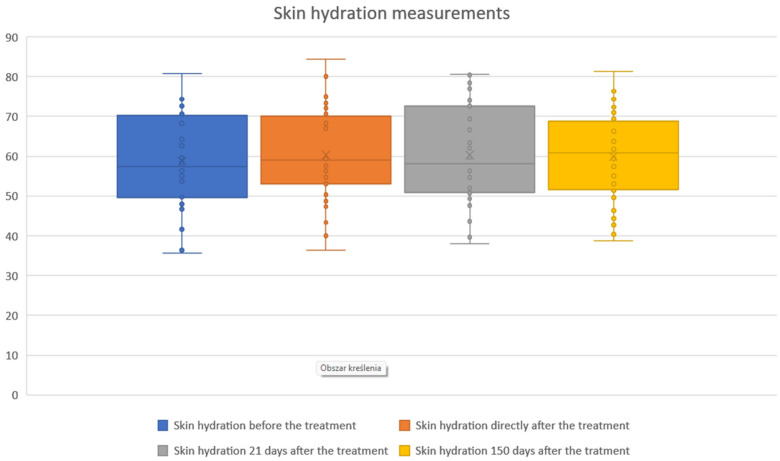
Skin hydration measurements before and after treatment. Boxplots illustrate skin hydration values assessed at baseline, immediately after treatment, 21 days post-treatment, and 150 days post-treatment. Median hydration levels and variability showed only modest fluctuations across the follow-up period, indicating that skin hydration remained relatively stable following the procedure.

**Figure 5:**
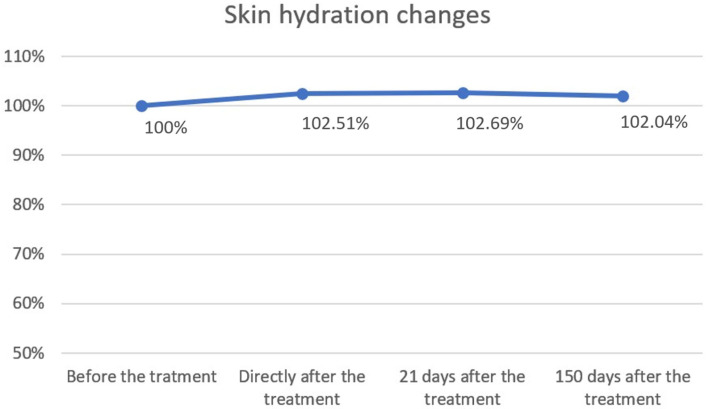
Skin hydration changes over time. Skin hydration levels were expressed as percentages relative to baseline (100%) and measured immediately after treatment, as well as 21 and 150 days post-treatment. A slight increase in hydration was observed directly after treatment (102.51%) and at day 21 (102.69%), followed by stabilization at day 150 (102.04%). Overall, hydration values remained close to baseline, indicating minimal long-term changes.

The study showed statistical significance and positive correlation of the effect on changes in the level of skin hydration at 5 and 21 days after the procedure. The effect was observed soon after the procedure, remains similar at 21 days, and slightly decreases by 150 days. The mean hydration values were as follows: baseline, 58.76; immediately after the procedure, 60.23 (2.51% increase from baseline); 21 days after the procedure, 60.33 (2.69% increase); and 150 days after the procedure, 59.96 (2.04% increase). The statistical analyses and corresponding graphical results are shown in the tables and figures below (Table 2; Figure 4 and Figure 5).

Healing time observation

**Figure 6:**
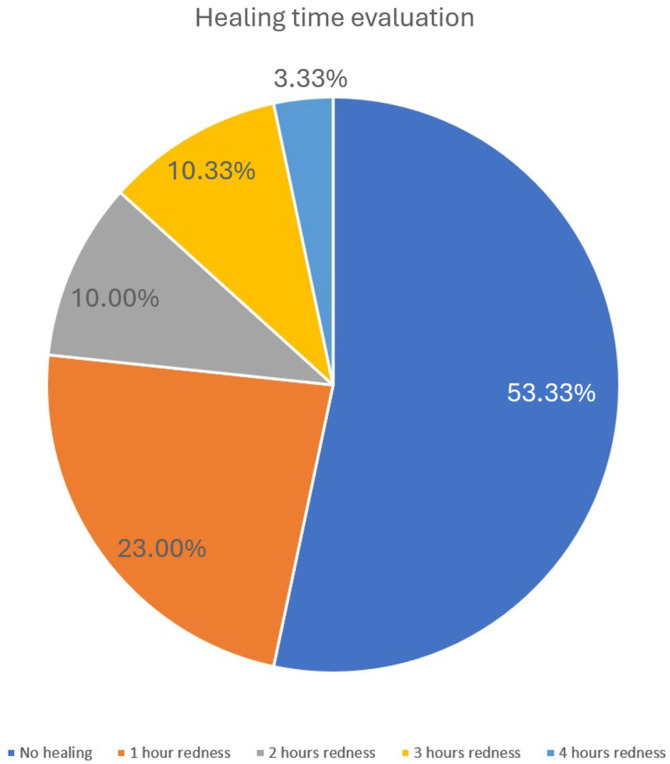
Healing time evaluation. The pie chart illustrates the distribution of healing times as assessed by the resolution of post-treatment erythema. More than half of the patients (53.33%) experienced no visible redness, while 23% showed redness lasting approximately 1 h. A smaller proportion reported erythema persisting for 2 h (10%), 3 h (10.33%), or up to 4 h (3.33%). These findings indicate that post-treatment reactions were generally mild and transient.

Post-treatment effects were limited to mild skin redness. No swelling or other noticeable skin reactions were reported by the participants. The majority of patients did not experience significant reddening; redness was reported by 14 participants (46.67%), lasting 1–4 h, with an average duration of 1.85 h among those affected. Results are illustrated in the chart below (Figure 6).

Patient’s satisfaction evaluation

Patients’ satisfaction with the procedure was generally high. The majority of participants reported improvements in skin tightening, reduction in fine wrinkles, skin texture, and skin color. On a five-point scale (1 = no improvement, 5 = very good improvement), most patients rated the outcomes as moderate to very good. The detailed distribution of responses for each assessed feature is summarized in the table below (Table 3).

**Table 3:** Patient satisfaction with treatment outcomes (self-assessment, N = X patients).

	Improvment				
Parameter:	Very Good	Good	Moderate	Poor	No
Skin tension	53.33%	33.33%	13.33%	–	–
Fine wrinkles	20.00%	50.00%	30.00%	–	–
Skin structure	–	–	16.67%	50.00%	33.33%
Skin color	–	30.00%	36.67%	23.33%	10.00%

**Figure 7:**
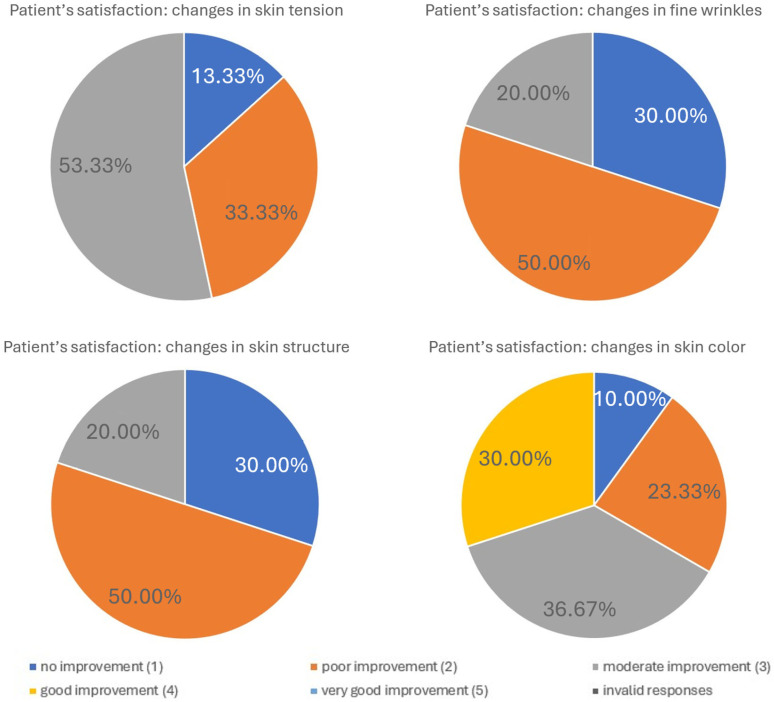
Patient satisfaction following treatment. Patient satisfaction levels were assessed using a standardized questionnaire administered after completion of the treatment series. The figure illustrates the distribution of responses across predefined satisfaction categories, highlighting the overall positive perception of the intervention. The majority of patients reported high satisfaction, while only a small proportion expressed neutral or low satisfaction. These results emphasize the favorable patient-reported outcomes associated with the procedure.

The graphical elaboration of the results is presented in the charts below (Figure 7).

Expert evaluation

An independent team of seven experts assessed the changes in skin tension, fine wrinkles, skin structure, and skin color based on patient photographs taken before and five months after the procedure. The results are summarized below (Table 4).

**Table 4:** Expert evaluation of clinical outcomes at 5 months (N = 7 experts).

	Improvement				
Parameter:	Very Good	Good	Moderate	Poor	No
Skin tension	39.52%	40.95%	18.57%	0.95%	–
Fine wrinkles	18.57%	43.33%	32.86%	5.24%	–
Skin structure	–	–	21.43%	47.14%	28.10%
Skin color	0.95%	27.62%	40.48%	19.05%	8.57%

**Figure 8:**
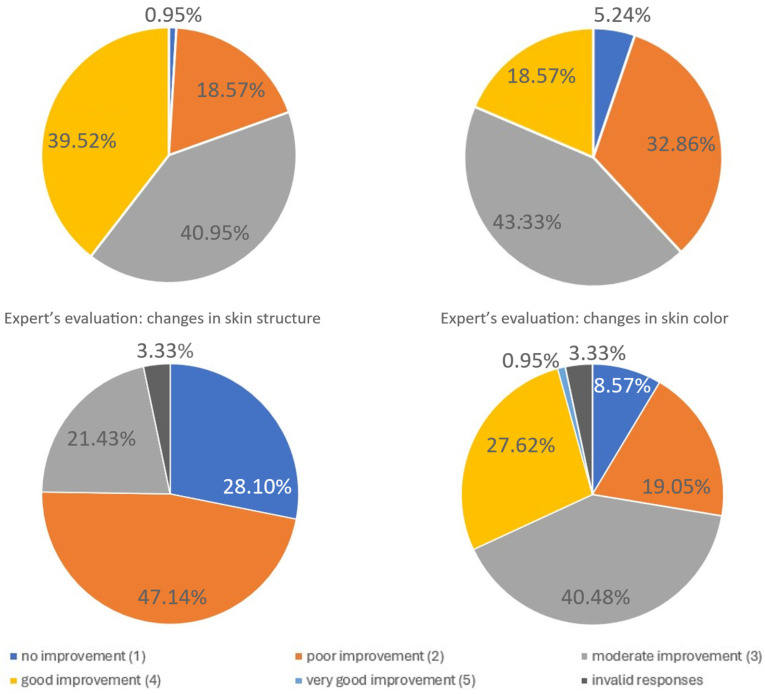
Expert evaluation of fine wrinkle changes. The figure summarizes expert assessments of changes in fine wrinkles following treatment. Independent evaluators rated the degree of improvement using predefined clinical criteria. The distribution of expert ratings demonstrates measurable improvements in wrinkle appearance, supporting the objective efficacy of the intervention in addition to patient-reported outcomes. Due to missing or invalid responses, the total percentage of categories may be <100%. Missing answers are represented on the pie chart as the dark grey segment.

The graphical elaboration of the results is presented in the charts below (Figure 8).

B. Results of histological assessment

Hematoxylin–eosin staining

**Figure 9:**
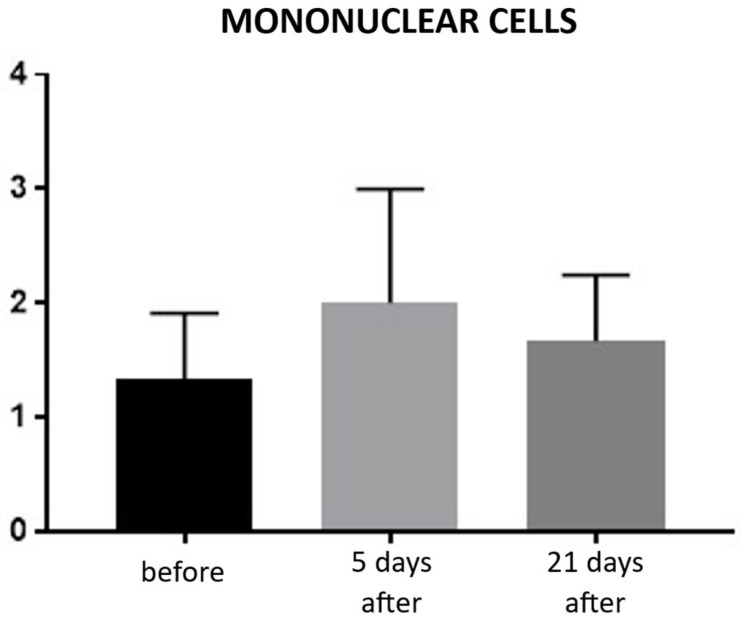
Inflammatory infiltrate in hematoxylin–eosin (H&E) staining. Bar graph showing the semi-quantitative assessment of inflammatory infiltrate at baseline (black), day 5 (light gray), and day 21 (dark gray) after treatment. No statistically significant differences were observed between time points.

**Figure 10:**
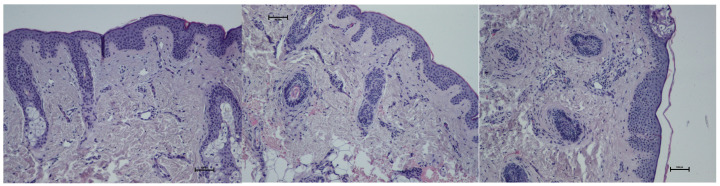
Representative hematoxylin–eosin staining of skin samples. Images are shown from left to right: immediately before treatment, 5 days after treatment, and 21 days after treatment. Staining was performed using an ECLIPSE E400 microscope (Nikon) at 20× magnification.

Evaluation of inflammatory infiltration revealed a baseline level of mononuclear cell presence before the procedure, with a mean score of 1.22. Five days after the treatment, an increase in inflammatory infiltration was observed, reaching a mean score of 2.0, reflecting the expected acute tissue response. By day 21, the inflammatory infiltration had decreased to a mean score of 1.66, indicating a partial resolution of the inflammatory response and progression toward tissue recovery (Figure 9; Table 5). Hematoxylin–eosin (H&E) staining was used to assess the overall tissue structure, including both cytoplasm and cell nuclei (Figure 10).

CD68 (monocytes and macrophages)

**Figure 11:**
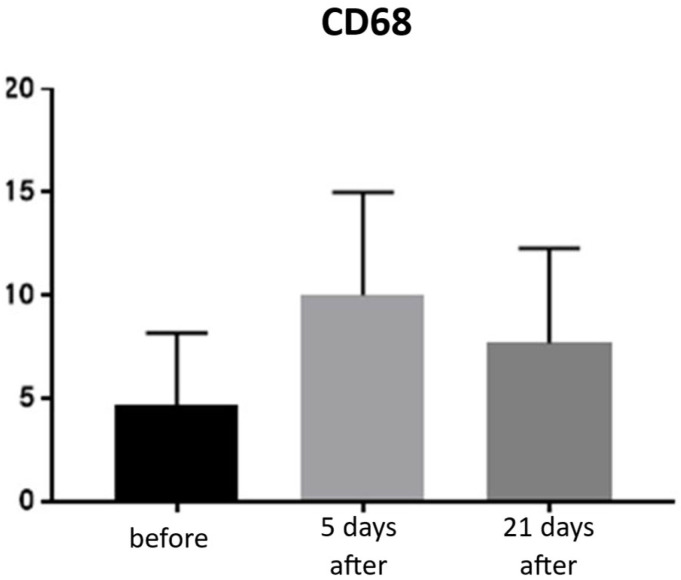
Monocyte and macrophage (CD68^+^) expression in skin samples. Bar graph showing the percentage of CD68^+^ cells within the inflammatory infiltrate at baseline (black), day 5 (light gray), and day 21 (dark gray) after treatment. No statistically significant differences were observed between time points.

**Figure 12:**
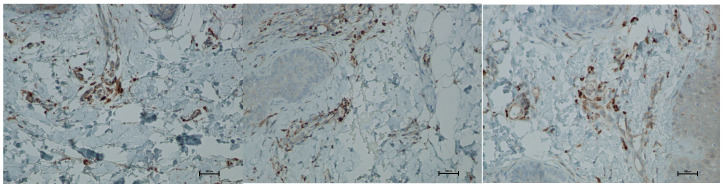
Representative CD68^+^ immunohistochemical staining of skin samples. Images are shown from left to right: immediately before treatment, 5 days after treatment, and 21 days after treatment. Captured using an ECLIPSE E400 microscope (Nikon) at 20× magnification.

Expression of CD68 proteins was assessed on a scale of 0–100% of inflammatory infiltrate cells. CD68 expression is associated with the presence of monocytes and macrophages, which are responsible for phagocytosing foreign substances. Prior to treatment, the average CD68 expression was 4.66% of inflammatory infiltrate cells. On day 5 post-procedure, this level increased to 10%, but by day 21, it decreased to 7.66% (Figure 11 and Figure 12; Table 6).

Lymphocytes T CD4^+^

**Figure 13:**
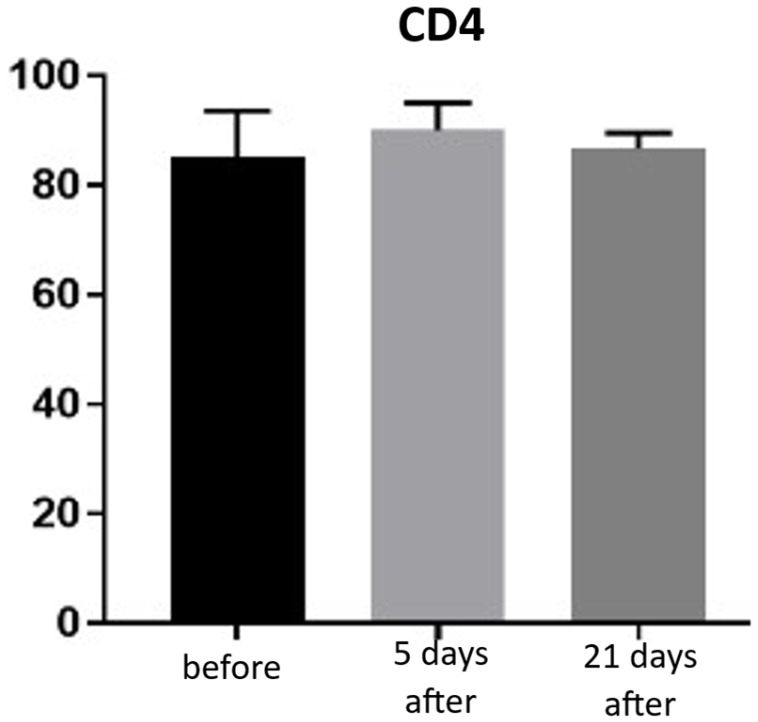
Th lymphocyte (CD4^+^) expression in skin samples. Bar graph showing the percentage of CD4^+^ cells within the inflammatory infiltrate at baseline (black), day 5 (light gray), and day 21 (dark gray) after treatment. No statistically significant differences were observed between time points.

**Figure 14:**
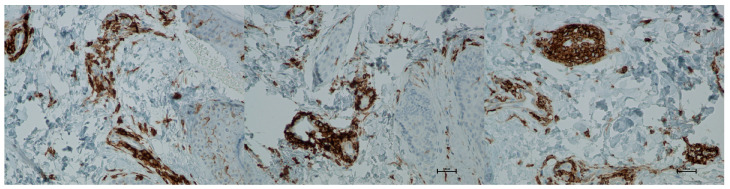
Representative CD4^+^ immunohistochemical staining of skin samples. Images are shown from left to right: immediately before treatment, 5 days after treatment, and 21 days after treatment. Captured using an ECLIPSE E400 microscope (Nikon) at 20× magnification.

Expression of CD4 proteins was assessed on a scale representing 0–100% of inflammatory infiltrate cells. CD4 proteins are associated with the presence of T-helper (Th) lymphocytes and are directly linked to the MHC class II antigen recognition system. Prior to treatment, the average CD4 expression was 85% of inflammatory infiltrate cells. On the 5th day after the procedure, this level increased to 90%. By day 21 post-procedure, the proportion of CD4-positive cells decreased slightly, reaching an average of 86.66% (Figure 13 and Figure 14; Table 7).

CD8^+^ (T lymphocytes)

**Figure 15:**
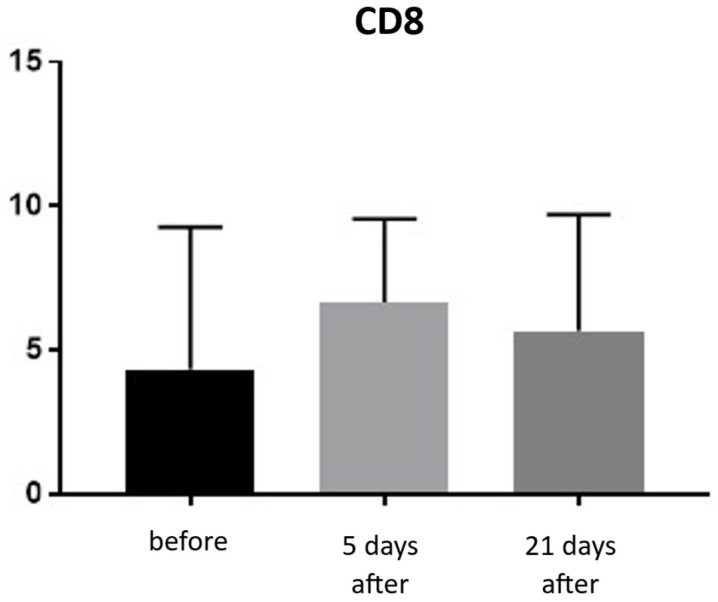
Tc lymphocyte (CD8^+^) expression in skin samples. Bar graph showing the percentage of CD8^+^ cells within the inflammatory infiltrate at baseline (black), day 5 (light gray), and day 21 (dark gray) after treatment. No statistically significant differences were observed between time points.

**Figure 16:**
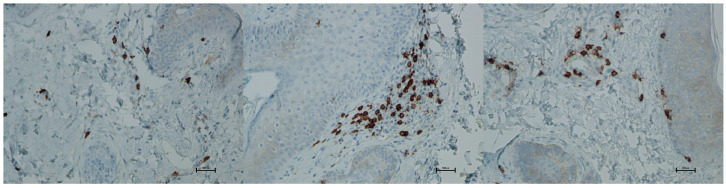
Representative CD8^+^ immunohistochemical staining of skin samples. Images are presented from left to right: immediately before treatment, 5 days after treatment, and 21 days after treatment. Captured with an ECLIPSE E400 microscope (Nikon) at 20× magnification.

Expression of CD8 proteins was assessed on a scale representing 0–100% of inflammatory infiltrate cells. CD8 proteins are associated with the presence of cytotoxic T lymphocytes (Tc lymphocytes/T-killer cells) and are directly linked to the MHC class I antigen recognition system. Before treatment, the average CD8 expression was 4.33% of inflammatory infiltrate cells. On the 5th day after the procedure, this level increased to 6.66%. By day 21 post-procedure, the proportion of CD8-positive cells decreased slightly, reaching an average of 5.66% (Figure 15 and Figure 16; Table 8).

CD20 (B lymphocytes)

**Figure 17:**
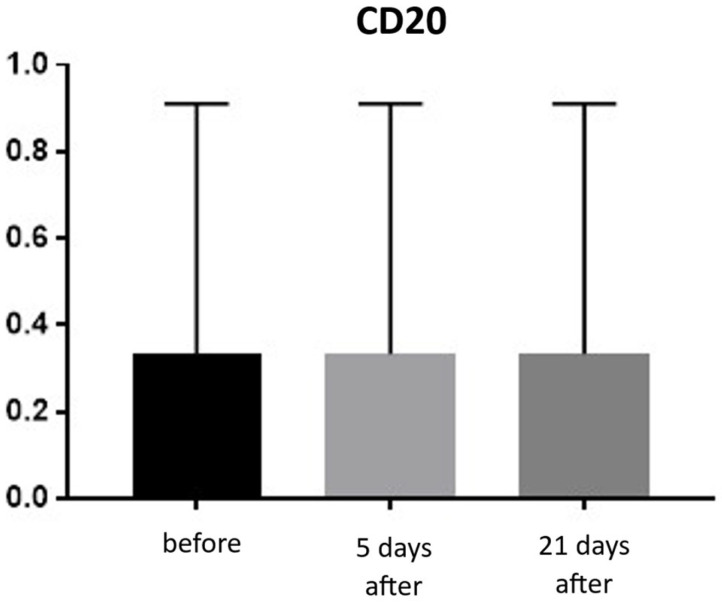
B lymphocyte (CD20^+^) expression in skin samples. Bar graph showing the percentage of CD20^+^ cells within the inflammatory infiltrate at baseline (black), day 5 (light gray), and day 21 (dark gray) after treatment. No significant differences were observed between time points.

**Figure 18:**
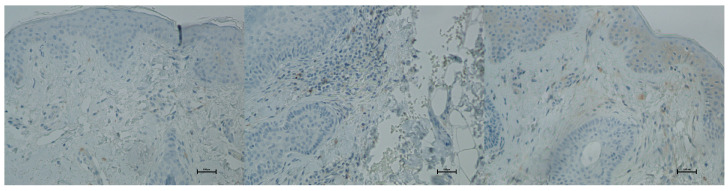
Representative CD20^+^ immunohistochemical staining of skin samples. Images are shown from left to right: immediately before treatment, 5 days after treatment, and 21 days after treatment. Captured with an ECLIPSE E400 microscope (Nikon) at 20× magnification.

Expression of CD20 is indicative of the presence of B lymphocytes, which are responsible for recognizing antigens independently of the major histocompatibility complex and for producing antibodies and cytokines. The presence of B lymphocytes may significantly influence the development of inflammatory reactions following procedures, including the administration of hyaluronic acid-based fillers. An increased proportion of these cells should be viewed negatively regarding both the safety and longevity of such procedures. Prior to treatment, the average proportion of CD20-positive cells was 0.33% of inflammatory cells, and no significant changes were observed over the subsequent 21 days (Figure 17 and Figure 18; Table 9).

CD31 (PECAM-1)

**Figure 19:**
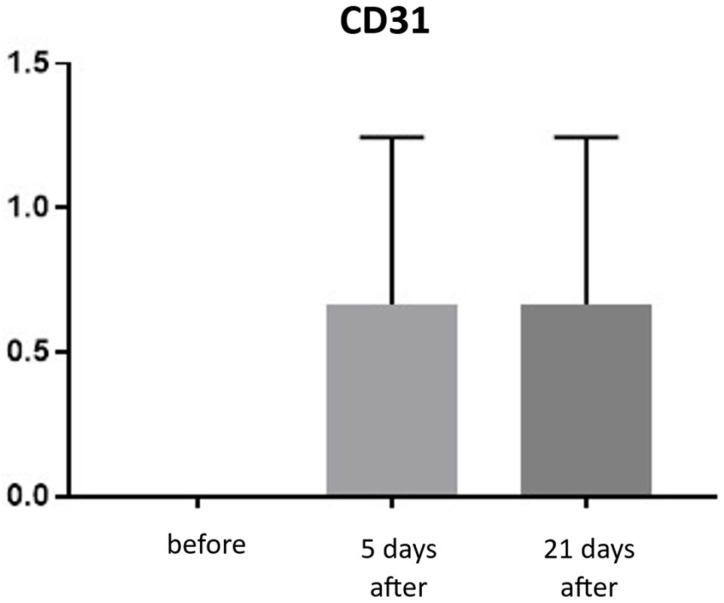
CD31 expression in skin samples. Bar graph showing mean CD31 expression levels (± SD) at day 5 (light gray) and day 21 (dark gray) after treatment. Expression was assessed using a semi-quantitative scoring scale ranging from 0 (no staining) to 2 (strong staining). No statistically significant differences were detected between time points.

**Figure 20:**
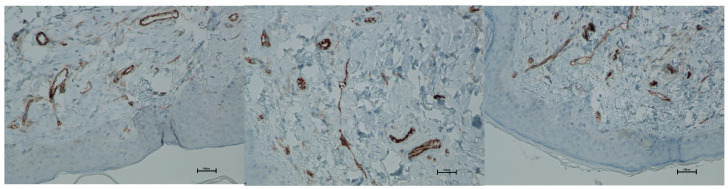
Representative CD31 immunohistochemical staining of skin samples. Images are presented from left to right: immediately before treatment, 5 days after treatment, and 21 days after treatment. Captured using an ECLIPSE E400 microscope (Nikon) at 20× magnification.

CD31 expression is associated with vascular endothelium and reflects angiogenesis, which, alongside neocollagenesis, is one of the expected effects of tissue revitalization procedures. The number of vessels was evaluated using the following scale: 0—few, thin-walled vessels; 1—increased number of vessels with slight wall thickening; 2—increased number of vessels with pronounced wall thickening. Before the procedure, the CD31 level was 0. At both 5 and 21 days post-treatment, the level increased to an average of 0.66 (Figure 19 and Figure 20; Table 10).

Vimentin (presence of fibroblasts)

**Figure 21:**
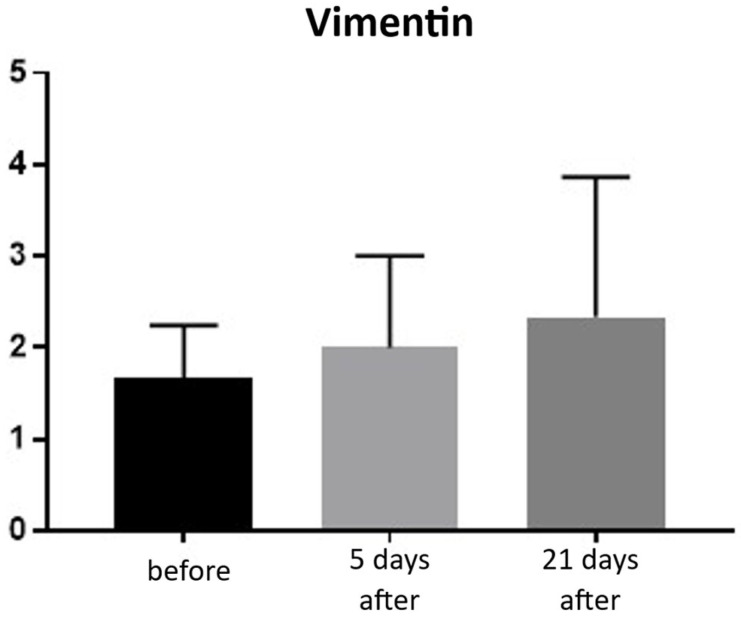
Vimentin expression in skin samples. Bar graph showing mean vimentin expression levels (± SD) at baseline (black), day 5 (light gray), and day 21 (dark gray) after treatment. Expression was evaluated using a semi-quantitative scoring scale ranging from 0 (no staining) to 4 (strong staining). No statistically significant differences were observed between time points.

**Figure 22:**
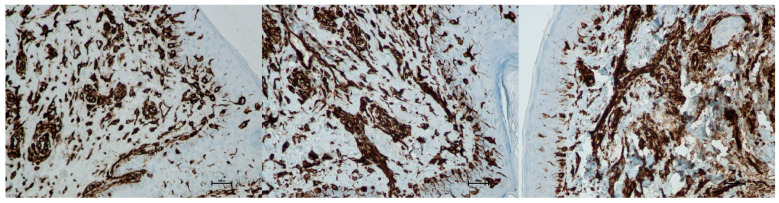
Representative vimentin immunohistochemical staining of skin samples. Images are shown from left to right: immediately before treatment, 5 days after treatment, and 21 days after treatment. Captured using an ECLIPSE E400 microscope (Nikon) at 20× magnification.

An increase in fibroblasts provides direct evidence of collagen production in the skin and ongoing tissue regeneration. Fibroblasts actively synthesize collagen, intercellular matrix fibers, and proteoglycans. The presence of fibroblasts was assessed using a semi-quantitative scale (0–4). Before treatment, the average fibroblast level was 1.66, increasing to 2.00 on day 5 and 2.33 by day 21 post-procedure. This corresponds to a 40% increase in fibroblasts at day 21 compared to baseline (Figure 21 and Figure 22; Table 11).

Autofluorescence analysis of unstained preparations

**Figure 23:**
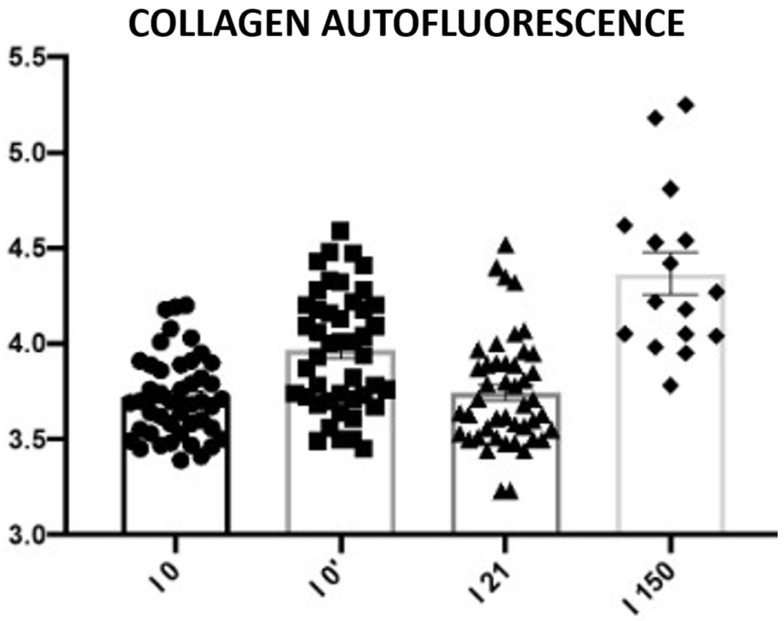
Subepidermal collagen autofluorescence. Representative images acquired using a 543 nm laser in combination with a 650 nm long-pass filter. Changes in collagen autofluorescence measured before the procedure (I0), immediately after the procedure (I0′), and on days 21 (I21) and 150 (I150) post-treatment. Circles, squares, triangles, and rhomboids represent individual data points within each group.

The aim of skin revitalization procedures, including infrared treatments, is to induce a measurable increase in collagen content. A statistically significant increase in collagen autofluorescence was observed in skin samples collected 5 days and 150 days after the procedure. No statistically significant changes were detected between samples taken before the procedure and those collected on day 21 post-treatment (Figure 23; Table 12).

## 4. Discussion

The present study demonstrates that wide-spectrum, long-pulse infrared irradiation induces both early histological responses and sustained clinical improvements, supporting its role as an effective non-ablative modality for facial rejuvenation. The combination of cutometric measurements, subjective evaluations, and histological analyses provides a coherent picture in which thermal dermal stimulation initiates a controlled inflammatory cascade, followed by fibroblast activation, collagen remodeling, and progressive enhancement of skin elasticity. These findings not only corroborate the established mechanisms of non-ablative dermal heating but also offer new cellular-level evidence for this specific infrared device.

Cutometric analysis revealed significant improvements in skin elasticity as early as 5 days post-treatment, with progressive enhancement observed at 21 and 150 days (Figure 2 and Figure 3; Table 1). This temporal pattern suggests that infrared-induced dermal heating initiates both immediate and sustained remodeling processes. Such long-term gains align with earlier studies, where infrared therapy was shown to stimulate neocollagenesis and matrix reorganization over several months [16,23]. Similar long-term improvements in elasticity were also reported by Lee et al., who demonstrated that far-infrared exposure significantly increased fibroblast collagen and elastin production and translated clinically into noticeable enhancement of skin tightness [23]. In contrast, skin hydration exhibited only modest improvements, peaking at 21 days and slightly declining by 150 days (Figure 4 and Figure 5; Table 2), suggesting that hydration changes may reflect transient epidermal barrier modulation rather than durable dermal restructuring.

Patients and independent experts reported high satisfaction, particularly regarding skin tightening and reductions in fine wrinkles, in line with the objective cutometric results (Figure 7 and Figure 8; Table 3 and Table 4). This agreement supports the clinical relevance of the treatment. However, noticeable discrepancies appeared in assessments of skin texture and color: patients tended to rate these improvements more positively than experts. These differences likely reflect distinct evaluation perspectives. Patients often perceive subtle, overall changes in skin appearance, whereas experts apply more stringent, standardized criteria focused on clearly measurable alterations. The more modest expert ratings for texture and color may also indicate that infrared thermolifting primarily affects deeper dermal structures, while superficial changes, such as pigmentation or fine surface irregularities—respond better to modalities like laser resurfacing or chemical peels [5,23,24]. This is consistent with findings by Lee et al., who reported minimal pigment improvement after far-infrared therapy [23], in contrast to the significant reductions demonstrated by Waibel and Schallen with the non-ablative fractional 1940 nm diode laser [24].

Histological analyses demonstrated a transient inflammatory infiltration, with its highest intensity observed at day 5 and resolving by day 21 (Figure 9 and Figure 10; Table 5). This mild and self-limiting inflammatory response is in line with the expected tissue reaction to sub-ablative thermal exposure, which serves as a stimulus for wound-healing pathways without inducing overt tissue damage [25]. Because biopsies were collected after the immediate acute phase (first 48–72 h), the infiltrate consisted mainly of mononuclear cells, supporting the focus on CD4^+^, CD8^+^, CD20^+^, and CD68^+^ markers rather than granulocyte-associated markers. Importantly, most changes in inflammatory markers (CD68, CD4, CD8, CD20), angiogenesis (CD31), and fibroblast activity (vimentin) were not statistically significant and should be interpreted as trends rather than definitive effects. These trends should also be interpreted in light of the small biopsy cohort, which may limit the sensitivity to detect subtle or heterogeneous tissue-level effects. The consistently low CD20^+^ levels suggest an absence of sustained humoral activation [26], while modest CD31 increases point toward early angiogenic activity [21]. A subtle and non-significant tendency toward increased CD68^+^ macrophages and CD4^+^ lymphocytes was observed, which may suggest the initiation of an early, remodeling-oriented immune response supporting matrix turnover and fibroblast activity (Figure 11, Figure 12, Figure 13 and Figure 14; Table 6 and Table 7). Overall, the findings indicate a mild, controlled post-acute remodeling response, though studies including earlier biopsy time points and larger biopsy cohorts would better characterize the acute granulocytic phase.

Fibroblast stimulation is a central mechanism of dermal remodeling, leading to synthesis of new collagen and extracellular matrix proteins that restore skin elasticity [27]. In our study, the increase in vimentin-positive fibroblasts from baseline to day 21 (Figure 21 and Figure 22; Table 11) should be viewed as a directional, rather than statistically conclusive, indicator of early cellular activation. More robust evidence of structural change was provided by collagen autofluorescence, which revealed significant increases at both 5 and 150 days (Figure 23; Table 12), demonstrating a durable remodeling response. The early rise likely reflects immediate collagen fiber contraction—a mechanism previously described by Zelickson et al. following controlled dermal heating—whereas the pronounced increase at 150 days is consistent with sustained neocollagenesis and matrix reorganization [28]. This biphasic pattern accords with Goldberg’s findings that non-ablative 1320 nm Nd:YAG irradiation induces new papillary dermal collagen and visible clinical improvement, and with other infrared and radiofrequency studies showing that collagen renewal can continue for months after sub-ablative stimulation [29]. In line with these observations, Kubik et al. reported marked increases in fibroblast density and up to a 72% improvement in elasticity at 150 days, supporting the concept of prolonged infrared-driven remodeling [16]. Similar gains in intradermal collagen density reported by Wunsch and Matuschka following red and near-infrared photobiomodulation further underscore that fibroblast-mediated collagen renewal is a shared hallmark of multiple light-based rejuvenation modalities [30].

Compared with ablative modalities such as CO_2_ or Er:YAG lasers, infrared therapy offers more modest improvements in epidermal features but demonstrates strong efficacy in dermal tightening with minimal downtime [8,31]. Ablative resurfacing remains the gold standard for treating texture irregularities and pigmentary changes, yet these benefits come at the cost of prolonged erythema, risk of post-inflammatory hyperpigmentation, and several days to weeks of recovery. In contrast, infrared thermolifting induces controlled sub-ablative heating of the dermis while preserving epidermal integrity, allowing patients to return to normal activity immediately after treatment. This favorable safety and tolerability profile was confirmed in our study, where no adverse events beyond transient erythema were observed. The short duration of post-treatment redness (mean 1.85 h) further underscores the minimally invasive nature of the procedure and aligns with previous reports describing infrared-based devices as consistently well tolerated, even in patients with darker phototypes or sensitive skin.

Although the biological effects of non-ablative thermal stimulation have been described for several infrared and mid-infrared modalities, detailed histological and autofluorescence data for this specific infrared device, and obtained under strictly monotherapy conditions without combination treatments have not been previously reported. Our study therefore provides the first structured, time-resolved evaluation of early and late remodeling responses for this platform, linking device-specific histological changes with objectively measured improvements in skin elasticity.

This study has several limitations. Histological analyses were performed in only three patients to minimize patient burden, which limits statistical robustness and generalizability. Biopsies were taken from the retroauricular area to avoid visible facial scarring—an ethical constraint in esthetic studies—but anatomical differences from facial skin may affect the applicability of these findings. The single-arm exploratory design allowed isolation of infrared-related tissue effects, yet the absence of a comparator group limits causal interpretation. The 150-day follow-up provides insight into medium-term remodeling, though long-term durability remains uncertain. Additionally, the ICDRG scale, used solely as a visual aid to standardize patient-reported erythema, is not validated for esthetic procedures and may introduce subjective bias. Future studies with larger biopsy cohorts, facial sampling when feasible, appropriate control arms, and extended follow-up will be essential to confirm and extend these preliminary results.

## 5. Conclusions

In summary, this study demonstrates that wide-spectrum, long-pulse infrared therapy induces favorable histological and clinical outcomes in patients with facial skin laxity and photodamage. The treatment was well tolerated, triggered transient but controlled inflammatory responses, stimulated fibroblast proliferation, promoted angiogenesis, and resulted in durable improvements in skin elasticity through collagen remodeling. While these findings support the regenerative potential of infrared energy, further validation in larger, controlled studies is needed to confirm long-term efficacy and to optimize treatment protocols for clinical practice.

## Figures and Tables

**Table 1:** Changes in skin elasticity before and after treatment.

Time Point	Mean ± SD	Δ vs. Baseline (Mean ± SD)	95% CI of Difference	*p*-Value
Baseline	58.92 ± 11.57	–	–	–
Day 5	68.37 ± 10.97	+9.44 ± 2.55	−10.40 to −8.49	<0.001
Day 21	72.11 ± 10.08	+13.19 ± 2.75	−14.22 to −12.16	<0.001
Day 150	73.27 ± 9.04	+14.35 ± 4.32	−15.96 to −12.73	<0.001

Mean ± SD values of skin elasticity are shown for baseline, day 5, day 21, and day 150 (N = 30). Differences vs. baseline, 95% confidence intervals, and *p*-values (paired *t*-test) are reported. All post-treatment measurements demonstrated significant improvements compared with baseline (*p* < 0.001). The symbol “–” indicates that no value is applicable or available for the baseline measurement.

**Table 2:** Skin hydration before and after treatment.

Time Point	Mean ± SD	Δ vs. Baseline (Mean ± SD)	95% CI of Difference	*p*-Value
Baseline	58.76 ± 12.59	–	–	–
Day 5	60.23 ± 12.70	+1.48 ± 3.30	−2.71 to −0.24	0.02
Day 21	60.33 ± 12.77	+1.58 ± 3.78	−2.99 to −0.17	0.03
Day 150	59.96 ± 11.61	+1.20 ± 4.18	−2.76 to 0.36	0.13

Mean ± SD values of skin hydration are shown for baseline, day 5, day 21, and day 150 (N = 30). Differences vs. baseline, 95% confidence intervals, and *p*-values (paired *t*-test) are reported. Small but statistically significant increases were observed at days 5 and 21, while changes at day 150 were not significant. The symbol “–” indicates that no value is applicable or available for the baseline measurement.

**Table 5:** Statistical analysis results for inflammatory infiltration.

Tukey’s Multiple Comparisons Test	Mean Diff.	95.00% CI of Diff.	Adjusted *p*-Value
0 vs. 5	−0.67	−2.52 to 1.18	0.669
0 vs. 21	−0.33	−2.18 to 1.52	0.936
5 vs. 21	0.33	−1.52 to 2.18	0.936

Mean differences (Mean Diff.), 95% confidence intervals (CI), and adjusted *p*-values are presented for pairwise comparisons between baseline (day 0), day 5, and day 21. No statistically significant differences were observed between time points (all adjusted *p* > 0.05).

**Table 6:** Statistical analysis results for monocytes and macrophages (CD68^+^) presence.

Tukey’s Multiple Comparisons Test	Mean Diff.	95.00% CI of Diff.	Adjusted *p*-Value
0 vs. 5	−0.67	−2.52 to 1.18	0.669
0 vs. 21	−0.33	−2.18 to 1.52	0.936
5 vs. 21	0.33	−1.52 to 2.18	0.936

Mean differences (Mean Diff.), 95% confidence intervals (CI), and adjusted *p*-values are presented for pairwise comparisons between baseline (day 0), day 5, and day 21. No statistically significant differences were observed between time points (all adjusted *p* > 0.05).

**Table 7:** Statistical analysis results for Th lymphocytes (CD4^+^) presence.

Tukey’s Multiple Comparisons Test	Mean Diff.	95.00% CI of Diff.	Adjusted *p*-Value
0 vs. 5	−5.00	−76.50 to 66.50	0.996
0 vs. 21	−1.67	−73.17 to 69.84	1.000
5 vs. 21	mar.33	−68.17 to 74.84	0.999

Mean differences (Mean Diff.), 95% confidence intervals (CI), and adjusted *p*-values are reported for pairwise comparisons between baseline (day 0), day 5, and day 21. No statistically significant differences were detected between groups (all adjusted *p* > 0.05).

**Table 8:** Statistical analysis results for T_c_ lymphocytes (CD8^+^) presence.

Tukey’s Multiple Comparisons Test	Mean Diff.	95.00% CI of Diff.	Adjusted *p*-Value
0 vs. 5	−2.33	−11.61 to 6.94	0.850
0 vs. 21	−1.33	−10.61 to 7.94	0.966
5 vs. 21	1.00	−8.28 to 10.28	0.985

Mean differences (Mean Diff.), 95% confidence intervals (CI), and adjusted *p*-values are reported for pairwise comparisons between baseline (day 0), day 5, and day 21. No statistically significant differences were detected between groups (all adjusted *p* > 0.05).

**Table 9:** Statistical analysis results for B lymphocytes (CD20^+^) presence.

Tukey’s Multiple Comparisons Test	Mean Diff.	95.00% CI of Diff.	Adjusted *p*-Value
0 vs. 5	0.00	−1.31 to 1.31	>0.999
0 vs. 21	0.00	−1.31 to 1.31	>0.999
5 vs. 21	0.00	−1.31 to 1.31	>0.999

Mean differences (Mean Diff.), 95% confidence intervals (CI), and adjusted *p*-values are reported for pairwise comparisons between baseline (day 0), day 5, and day 21. No differences were detected between time points (all adjusted *p* > 0.999).

**Table 10:** Statistical analysis results for CD31 expression.

Tukey’s Multiple Comparisons Test	Mean Diff.	95.00% CI of Diff.	Adjusted *p*-Value
0 vs. 5	−0.67	−1.73 to 0.40	0.264
0 vs. 21	−0.67	−1.73 to 0.40	0.264
5 vs. 21	0.00	−1.07 to 1.07	>0.999

Mean differences (Mean Diff.), 95% confidence intervals (CI), and adjusted *p*-values are reported for pairwise comparisons between baseline (day 0), day 5, and day 21. No statistically significant differences were observed between time points.

**Table 11:** Statistical analysis results for vimentin expression.

Tukey’s Multiple Comparisons Test	Mean Diff.	95.00% CI of Diff.	Adjusted *p*-Value
0 vs. 5	−0.33	−2.95 to 2.28	0.976
0 vs. 21	−0.67	−3.28 to 1.95	0.845
5 vs. 21	−0.33	−2.95 to 2.28	0.976

Mean differences (Mean Diff.), 95% confidence intervals (CI), and adjusted *p*-values are shown for pairwise comparisons between baseline (day 0), day 5, and day 21. No statistically significant differences were found between time points (all adjusted *p* > 0.05).

**Table 12:** Statistical analysis results for collagen autofluorescence.

Tukey’s Multiple Comparisons Test	Mean Diff.	95.00% CI of Diff.	Adjusted *p*-Value
0 vs. 5	−0.24	−0.41 to −0.08	0.0007 (***)
0 vs. 21	−0.02	−0.18 to 0.14	0.982 (ns)
0 vs. 150	−0.65	−0.87 to −0.42	<0.0001 (****)
0 vs. 5	−0.24	−0.41 to −0.08	0.0007 (***)
0 vs. 21	−0.02	−0.18 to 0.14	0.982 (ns)
0 vs. 150	−0.65	−0.87 to −0.42	<0.0001 (****)

Mean differences (Mean Diff.), 95% confidence intervals (CI), and adjusted *p*-values (with significance levels indicated as ns—not statistically significant, *** *p* < 0.001, **** *p* < 0.0001) are reported for pairwise comparisons between baseline (day 0), day 5, day 21, and day 150. Significant changes in autofluorescence were detected at day 5 and day 150 compared with baseline, and between several follow-up points, reflecting dynamic collagen remodeling over time.

## Data Availability

The data presented in this study are available on request from the corresponding author. The data are not publicly available due to privacy restrictions.
