# Correction: Kubik et al. An Infrared Energy Device for the Treatment of Facial Skin Aging. *Biomedicines* 2025, *13*, 2878

**DOI:** 10.3390/biomedicines14010133

**Published:** 2026-01-09

**Authors:** Paweł Kubik, Wojciech Gruszczyński, Aleksandra Pawłowska, Maciej Malinowski, Brygida Baran, Agnieszka Pawłowska-Kubik, Łukasz Kodłubański, Bartłomiej Łukasik

**Affiliations:** 1K-LAB Badania i Rozwoj, 81-312 Gdynia, Poland; wojciech.gruszczynski@k-lab.com.pl (W.G.); aleksandra.pawlowska@k-lab.com.pl (A.P.); maciejmal@gmail.com (M.M.); agnieszkapawl@wp.pl (A.P.-K.); 2Medical Department, Matex Lab Switzerland SA, 1228 Geneve, Switzerland; brygida.baran@neauvia.com (B.B.); bartlomiej.lukasik@neauvia.com (B.Ł.); 3Department of Human Rights and Intellectual Property Law, University of Gdansk, 80-309 Gdansk, Poland; lukasz.kod@gmail.com

## Figure Legend

In the original version of the manuscript [1], the caption of Figure 1 did not include full copyright attribution to the original author. The caption has therefore been updated to include the statement “© Radosław Śpiewak, published with kind permission” and a reference to the source article. The correct Figure 1 caption appears below. This correction was approved by the Academic Editor. The original publication has also been updated.**Figure 1.** **Visualization of the International Contact Dermatitis Research Group (ICDRG) scale provided to participants.** To enhance scientific clarity, dermatological terminology has been standardized in accordance with the ICDRG guidelines. **Negative reaction:** no visible or palpable change. (?) **Doubtful reaction:** subtle erythema, non-palpable erythematous macule; such a response is generally not considered evidence of sensitization. (+) **Weak reaction:** palpable erythema suggesting mild edema or infiltration, with or without small papules; no vesicles. (++) **Strong reaction:** pronounced edema and infiltration with papules and distinct vesicles. (+++) **Extreme reaction:** coalescing vesicles or bullae, occasionally accompanied by superficial erosions. (IR) **Irritant reaction:** non-allergic response characterized by shiny or dry skin, erythema, or fine papules. © Radosław Śpiewak, published with kind permission. Reproduced from: Śpiewak R. Patch testing for contact allergy and allergic contact dermatitis. *Open Allergy Journal* **2008**, *1*, 42–51 [17].

## References

Add the following reference to the Reference section of the original publication:17.Śpiewak, R. Patch testing for contact allergy and allergic contact dermatitis. *Open Allergy J.*
**2008**, *1*, 42–51. https://doi.org/10.2174/1874838400801010042.

With this correction, the order of some references (references 17–32) has been adjusted accordingly. The authors state that the scientific conclusions are unaffected. This correction was approved by the Academic Editor. The original publication has also been updated.
